# Contribution to the Study of Puerperal Septicæmia

**Published:** 1873-06

**Authors:** 


### Contribution to the Study of Puerperal Septicæmia.

M. A. D'Espine has published a most elaborate and instructive memoir on this subject, extending through four numbers of the *Archives Generales de Medecine.* The following are given by him in his concluding article, in the October number of the above journal, as the conclusions he feels himself justified in drawing from his investigations:

1. Puerperal septicæmia consists of a series of symptoms, the severity of which is in proportion to the quantity of septic matter absorbed by breaches of surface in the utero-vaginal canal.

2. These symptoms are not peculiar to the puerperal state, and ought not to be classed with those produced by septicæmia in the wounded and in animals.

3. The source of puerperal septicæmia is always the uterus or vagina; and all causes which prevent the healing of the bared interior of the uterus, or which favor the production of septic matter in its neighborhood, have an important action in its production.

4. The most common channel of absorption is through the lymphatics, and its passage through them can generally, but not always, be traced by lymphangitis.

5. Peritonitis is the result of the conveyance of septic matters through the lymphatics of the uterus, and it may be compared to the local inflammations which develop round infected wounds.

6. The effect of septic absorption is to develop congestions and inflammations in internal organs, chiefly in the lungs, kidneys and intestines; subserous ecchymoses and interstitial apoplexy; internal and external inflammations, which localize themselves in the neighborhood of the serous membranes; *during life,* these actions are recognized by fever, diarrhœa, pulmonary congestion, epistaxis, and often by fugitive cutaneous eruptions.

7. Milk fever has no existence; febrile action in the first week after delivery almost always depends on absorption of lochia through slight abrasions or lacerations of the utero-vaginal canal. It may continue for some weeks should the uterus not be firmly contracted, or should the lochia be fetid. In the latter case ulcerations, through which absorption takes place, may almost always be found either on the cervix or in the vagina.

8. These slighter affections are often, but not always, accompanied by angioleucitis and slight perimetritis. When the septic poison continues long, we may have consumption and death (*phthisie septique*).

9. Puerperal *pyœmia* is a complication of septicæmia, and is almost always accompanied by the presence of pus in the veins of the uterus.

It is a comparatively rare occurrence, aud probably depends on septic embola. Metastatic visceral abscesses are secondary to it, while almost all the inflammations of the cellular tissue and of the articulations depend on lymphatic infection, and are not embolic in their origin.—*Am. Jour. Med.*

